# Mutualism-disrupting allelopathic invader drives carbon stress and vital rate decline in a forest perennial herb

**DOI:** 10.1093/aobpla/plv014

**Published:** 2015-02-27

**Authors:** Nathan L. Brouwer, Alison N. Hale, Susan Kalisz

**Affiliations:** Department of Biological Sciences, University of Pittsburgh, Pittsburgh, PA 15260, USA

**Keywords:** Allelochemicals, *Alliaria petiolata*, carbon stress/carbon starvation, *Maianthemum racemosum*, mutualism disruption, root fungal symbiont, species invasion, vital rates

## Abstract

Invasive plants can negatively affect belowground processes and alter soil microbial communities. For native plants that depend on soil resources from root fungal symbionts (RFS), invasion could compromise their resource status and subsequent ability to manufacture and store carbohydrates. Herbaceous perennials that depend on RFS-derived resources dominate eastern North American forest understories. Therefore, we predict that forest invasion by *Alliaria petiolata*, an allelopathic species that produces chemicals that are toxic to RFS, will diminish plant carbon storage and fitness. Over a single growing season, the loss of RFS could reduce a plant's photosynthetic physiology and carbon storage. If maintained over multiple growing seasons, this could create a condition of carbon stress and declines in plant vital rates. Here we characterize the signals of carbon stress over a short timeframe and explore the long-term consequence of *Alliaria* invasion using *Maianthemum racemosum*, an RFS-dependent forest understory perennial. First, in a greenhouse experiment, we treated the soil of potted *Maianthemum* with fresh leaf tissue from either *Alliaria* or *Hesperis matronalis* (control) for a single growing season. *Alliaria*-treated plants exhibit significant overall reductions in total non-structural carbohydrates and have 17 % less storage carbohydrates relative to controls. Second, we monitored *Maianthemum* vital rates in paired experimental plots where we either removed emerging *Alliaria* seedlings each spring or left *Alliaria* at ambient levels for 7 years. Where *Alliaria* is removed, *Maianthemum* size and vital rates improve significantly: flowering probability increases, while the probability of plants regressing to non-flowering stages or entering prolonged dormancy are reduced. Together, our results are consistent with the hypothesis that disruption of a ubiquitous mutualism following species invasion creates symptoms of carbon stress for species dependent on RFS. Disruption of plant–fungal mutualisms may generally contribute to the common, large-scale declines in forest biodiversity observed in the wake of allelopathic invaders.

## Abstract (precis)

Most plants form mutually beneficial relationships with microorganisms in their roots, especially fungi. Invasive plants can release substances toxic to other species known as allelochemicals. Allelochemicals from the invasive species garlic mustard can inhibit beneficial soil fungi, thereby disrupting the plant-fungal mutualism. We demonstrate that treatment with garlic mustard leaves reduces the ability of a native plant, False Solomon's seal, to store carbohydrates. Additionally, we demonstrate that weeding garlic mustard from forest plots allows this native to grow larger, flower more frequently, and enter long-term dormancy less often relative to plots where garlic mustard occurs.

## Introduction

The majority of flowering plant species form mutualisms with root fungal symbionts (RFS) such as arbuscular mycorrhizal fungi (AMF; 74 % of angiosperms; Brundrett 2009) and dark septate endophytes (DSE; ≥600 species; Jumpponen and Trappe 1998). Arbuscular mycorrhizal fungi and DSE live inside plant roots and deploy hyphae outside the root that increase water, nitrogen, phosphorus and other soil nutrients' availability to their plant partner (Smith and Read 2008; Newsham 2011). The RFS receive a substantial fraction of the plant partner's fixed carbon (for AMF up to 20 %; Smith and Read 2008).

Recent work highlights how anthropogenic changes in the environment, such as invasion, can negatively affect mutualisms (Tylianakis *et al.* 2008; Kiers *et al.* 2010). Invasive species can impact belowground processes and directly or indirectly alter soil microbial communities, including RFS. Mechanisms through which belowground impacts can occur (summarized in part by Wolfe and Klironomos 2005) include alterations in the quality, quantity and timing of litter inputs and subsequent changes in soil nutrient status (reviewed by Ehrenfeld 2003), direct changes to soil nutrient status through novel nutrient fixation strategies by the invader (e.g. Vitousek and Walker 1989), mutualist degradation (Vogelsang and Bever 2009) and allelopathy (e.g. Callaway *et al.* 2008; Grove *et al*. 2012). Specifically, allelochemicals can act as novel weapons that are directly toxic to plants or act indirectly on their associated microbes (Callaway and Ridenour 2004; Weir *et al.* 2004).

The invasion of North American forests by *Alliaria petiolata* (Brassicaceae, garlic mustard) is an emerging model system for investigations of allelopathic effects on belowground processes (Rodgers *et al.* 2008*a*). This species produces a suite of allelochemicals (Vaughn and Berhow 1999; Cipollini and Gruner 2007) that are toxic to RFS (Roberts and Anderson 2001; Stinson *et al.* 2006; Koch *et al.* 2011) even at low concentrations (Callaway *et al*. 2008; Cantor *et al.* 2011). Field studies document that areas infested with *Alliaria* exhibit shifts in soil fungal community composition with frequent reductions in AMF species richness (Burke *et al*. 2011; Lankau 2011*a*; Lankau *et al.* 2014), declines in total soil hyphal abundances (Cantor *et al.* 2011; Koch *et al*. 2011) and changes in the within-root community of AMF-dependent plants (Burke 2008; Bongard *et al.* 2013). Together, these studies suggest that within *Alliaria-*invaded ecosystems the function of the mutualistic fungal community can be compromised and that these changes contribute to *Alliaria*'s invasive success.

Herbaceous perennials dominate the temperate forest understories that *Alliaria* invades and these species as a group are typically highly- to obligately-dependent on RFS (Brundrett and Kendrick 1988; Whigham 2004). The fact that temperate forest soils are strongly resource limited (Whigham 2004; Gilliam 2014) likely drives the obligate nature of the relationship for many understory herbaceous perennials. Typically these species are slow growing (Gilliam 2014), exhibit high rates of RFS colonization (e.g. Brundrett and Kendrick 1988; Boerner 1990; Burke 2008) and have long-lived arbuscules (Brundrett and Kendrick 1990). Many also lack fine roots or root hairs (e.g. LaFrankie 1985) perhaps because their associated RFS hyphae fulfil this soil resource-gathering role. Since resources supplied by RFS are intimately tied to many plant metabolic functions (Schweiger *et al.* 2014), disruption of soil mutualisms is expected to severely limit the physiological rates of forest species (Hale *et al.* 2011). In the absence of RFS, plants generally exhibit reduced photosynthetic rates (Allen *et al.* 1981; Wright *et al.* 1998; Zhu *et al.* 2011) and subsequent carbon stress can curb their ability to carry out carbon-demanding functions such as growth (Lu and Koide 1994) and flowering (Koide *et al.* 1994).

Carbon stress is the reduction of a plant's pool of total non-structural carbohydrates (NSCs) (*sensu* Anderegg *et al.* 2012). In herbaceous perennials, chronic carbon stress can alter key vital rates including survival (Gremer and Sala 2013), flowering (Crone *et al.* 2009) and prolonged dormancy (Gremer *et al.* 2010). Invaders like *Alliaria* that alter the soil environment and essential RFS functions could induce carbon stress or ‘carbon starvation’ (*sensu* McDowell *et al.* 2008), ultimately diminishing the stability of populations of RFS-dependent native species.

Our prior experiments on the RFS-dependent understory perennial, *Maianthemum racemosum* (Ruscaceae, false Solomon's seal) confirm the dramatic physiological consequences of short-term RFS disruption by *Alliaria*'s allelochemicals. Key physiological traits including stomatal conductance, which is known to be highly dependent on RFS colonization (Augé *et al*. 2014), and photosynthetic rate both significantly declined in plants exposed to fresh *Alliaria* leaf litter (Hale *et al.* 2011). Soil respiration, to which fungi are the primary contributors (Anderson and Domsch 1975), was also reduced with *Alliaria* treatment. Importantly, in field plots invaded by *Alliaria* and in pot experiments with an *Alliaria* litter treatment, we demonstrated significant declines in the abundance of soil fungal hyphae relative to controls (37 % decline, Cantor *et al.* 2011; 29–38 % decline, A. N. Hale *et al.*, submitted for publication). Together these data strongly support the idea that the observed physiological declines are driven by the inhibition of the RFS hyphal network in the soil (Hale *et al.* 2011).

Here we explore how the physiological stress of RFS-mutualism disruption in *Alliaria*-invaded forests could result in performance declines in an RFS-dependent forest perennial across two time scales. First, we ask: Given that *Alliaria*'s allelochemicals cause detectable shifts in the soil fungal community and alternative plant physiological rates, do they also cause declines in carbon storage in plants within a single growing season? In a greenhouse experiment we show that *Alliaria-*treated *Maianthemum* store significantly less carbon in their rhizome over one growing season relative to controls. Second, to determine the potential for short-term effects to scale up over time and affect population processes, we conducted a 7-year field experiment in an *Alliaria*-invaded forest in which *Alliaria* was weeded or left at ambient levels. We test whether *Maianthemum* exhibit lower growth rates consistent with carbon stress in the *Alliaria*-ambient plots. We also ask if *Alliaria* reduces size-based vital rates of *Maianthemum* and if so, how quickly these changes occur. We show that where *Alliaria* is present, *Maianthemum* have suppressed growth and vital rates relative to adjacent plot where *Alliaria* is removed.

## Methods

### Greenhouse study: assessing potential for carbon stress

The greenhouse study was conducted during the summer of 2010 in the greenhouse facilities at the University of Pittsburgh. In May, we obtained bare-root adult *Maianthemum* plants (*N* = 42) from a native plant nursery (Prairie Moon Nursery, Winona, MN, USA). Rhizomes ranged in size from 6.7 to 39.7 g fresh weight. We potted each rhizome in a 3 : 1 mixture of autoclaved Fafard potting soil and Turface. We inoculated plants with RFS by adding 150 g of field soil collected from areas adjacent to *Maianthemum* plants at our experimental field site (see details below). Pots were then placed in the greenhouse and watered every 2–3 days for 1 month, allowing the plants to complete stem elongation and establish the RFS mutualism.

In June, we assigned each plant to either an *Alliaria* treatment or a control treatment. To control for potential differences in initial carbohydrate status due to differences in plant age and/or size (e.g. Olano *et al.* 2006), we stratified the randomized assignment of rhizomes into the treatments to ensure that mean rhizome mass was the same in the *Alliaria* and control treatments. Plants in the *Alliaria* treatment were then exposed to *Alliaria* allelochemicals by placing 25 g of fresh *Alliaria* leaf tissue collected from a population with a recent history of invasion (<20 years) on top of the soil. When these plants were watered, the glucosinolates leached out of the *Alliaria* leaves and into the soil (A. N. Hale *et al.*, submitted for publication). As in previous experiments (Hale *et al.* 2011), plants in the control treatment received 25 g of fresh *Hesperis matronalis* (dame's rocket; Brassicaceae) leaf tissue. Like *Alliaria*, *Hesperis* is an invasive mustard in eastern North America (Leicht-Young *et al*. 2012). While *Hesperis* produces some glucosinolates (Larsen *et al.* 1992), RFS hyphae and vesicles have been observed within its root system (DeMars and Boerner 1995), indicating that *Hesperis* chemicals are less toxic to RFS than *Alliaria*. In the field, the high mortality rates of *Alliaria* seedlings and rosettes throughout the year (Davis *et al.* 2006) and the mortality of adults in the summer (Anderson *et al.* 1996) likely result in a sustained supply of allelochemicals into the soil. Thus, we re-applied fresh leaf tissue in both treatments every 2 weeks until the end of August to simulate a season-long supply of *Alliaria* allelochemicals.

We destructively harvested plants three times during the growing season (9 July, 6 August and at senescence) to assess the effect of the treatments on the carbohydrate status. For the last time point, we classified plants as being senesced when 40 % of the leaf tissue had yellowed and photosynthetic rates were <1.0 μmol m^−2^ s^−1^. Details of the leaf gas exchange protocol for *Maianthemum* can be found in Hale *et al*. (2011). To harvest the plants, we carefully clipped the shoot and roots away from the rhizome. We also stained the roots of a subset of plants per treatment following Brundrett *et al*. (1984) to confirm RFS colonization. We then weighed the rhizome and immediately flash-froze it in liquid nitrogen. We stored samples at −80 °C until they could be lyophilized and ground. We followed the protocol of Zuleta and Sambucetti (2001) to analyse rhizome inulin (storage carbohydrate) and sucrose (mobile carbohydrate) content via high-performance liquid chromatography (HPLC). [Note: Starch is not present in the rhizome of *Maianthemum* (A. N. Hale *et al.*, submitted for publication).] In brief, a 0.03 g dried sample for each plant is boiled while stirring with a magnetic stir bar. Once samples cool to room temperature, they are filtered through a 0.20 μm filter, and run on HPLC (Aminex HPX-87C anion-exchange column, deionized water at 85 °C was set as the mobile phase with a flux rate of 0.6 mL min^−1^). Standards are used (inulin from dahlia tubers, Sigma-Aldrich; sucrose, Sigma-Aldrich) to confirm the identity of the sample peaks and to create standard curves to determine inulin and sucrose concentrations. Here, we express inulin and sucrose concentrations as a percentage of the HPLC dry sample mass. We also sum each plant's inulin and sucrose content to determine total NSC concentration (%).

To explore the effect of our treatments on rhizome carbohydrate status, we use a multivariate analysis of covariance (MANCOVA). Following a significant MANCOVA, individual ANCOVA tests are conducted for inulin, sucrose and total NSC. For all models, we include harvest date as a main effect because rhizome carbohydrate concentration varies over the growing season in perennial herbs (e.g. Lapointe 1998; Wyka 1999; Kleijn *et al.* 2005). We also include initial plant mass as a covariate to account for differences in carbohydrate storage that are related to plant size/age (MANCOVA model: total NSC + inulin + sucrose = treatment + harvest date + initial plant mass; ANCOVA models: carbohydrate = treatment + harvest date + initial plant mass). We calculate least squares means and standard errors for all ANCOVA models with a significant (*P* < 0.05) treatment effect. All analyses were conducted in SAS (v. 9.3, SAS Institute, Cary, NC, USA).

### Field study: measuring impacts on vital rates of native plant populations

#### Study site

Our experimental plots are located in a beech-maple forest in southwest Pennsylvania [Trillium Trail Nature Reserve (hereafter TT), Allegheny County, PA, USA: 40°52′01.40″N; 79°90′10.75″W] with a rich herbaceous perennial understory flora (Knight *et al.* 2009). Based on previous work at TT (Burke 2008) and other temperate deciduous forests (e.g. Brundrett and Kendrick 1988), we estimate that 73 % of TT herbaceous perennials are AMF-dependent (Hale *et al.* 2011). We detected *Alliaria* allelochemicals in the soil of TT in concentrations that are toxic to AMF spores in lab assays (Cantor *et al.* 2011). Additionally, we showed that in soils where *Alliaria* occurs at TT, the density of fungal hyphae is lower (Cantor *et al*. 2011) and the fungal community composition shifts (Burke *et al*. 2011) relative to paired, non-invaded areas. *Maianthemum* plants collected at TT are heavily colonized by RFS, but their intra-root AMF community is significantly altered where *Alliaria* is present (Burke 2008). These results motivate further investigation of mutualism disruption by *Alliaria* in understanding mechanisms driving native plant performance declines.

#### Field experiment

We collected data on naturally occurring individuals of *M. racemosum* within six 14 × 14 m plots in TT from 2003 through 2013. Our six plots are split in half longitudinally so that each contains two experimental treatments: *Alliaria* removal (= low or no allelochemicals) or *Alliaria* present at ambient levels (= allelochemicals present). Annual removal of *Alliaria* from half of each plot (i.e. a 14 × 7 m area) began in spring 2006, ∼15 years after *Alliaria* became established at this site (L. Smith, pers. comm.) This time frame for TT invasion coincides with the estimated *Alliaria* invasion history in the region that indicates that this invader has been present locally for <25 years (Lankau *et al*. 2009). We remove *Alliaria* concurrent with the onset of emergence of the perennial herb community. *Alliaria* individuals are removed as tiny seedlings, minimizing disturbance to the soil and other plants. Removed plants are discarded off site. In June of each year prior to *Alliaria* seed dispersal we erect a barrier at the border of the two treatments to block seed dispersal from the ambient into the *Alliaria* removal treatment. All *Maianthemum* plants emerging in the plots are permanently tagged and have annually been scored for individual size, stage (i.e. seedling, non-flowering, flowering and dormant) and deer browse status. Prior to initiation of the *Alliaria* removal treatment in 2006 there was no difference in *Alliaria* per cent cover between the plots (*χ*^2^ = 0.11, *P* = 0.74) or total per cent cover of all species (*χ*^2^ = 0.038, *P* = 0.85).

#### Plant vital rates

We assess the effect of *Alliaria* removal on *Maianthemum* growth and three vital rates: annual flowering frequency, retrogression of flowering plants to non-flowering the following year and the frequency of prolonged vegetative dormancy (Shefferson 2009). We test for differences using data collected prior to the implementation of the removal treatment (2003–06) and after the removal treatment began (2007–13). All models have the general form: response variable = treatment + year + treatment × year. To estimate differences in growth rate, we investigate the differences in average size between treatments for the initial cohort of plants first observed when the experiment began in 2003. The mean size of this cohort is estimated with a linear mixed model for each year since 2006 (Zuur *et al.* 2009). We model log(plant size) to improve normality of the residuals.

Annual flowering frequencies are modelled using a logistic mixed model. Retrogression frequencies were modelled without random effects for the years 2008–13 because of limited sample size. Our retrogression model, stated in terms of probability, is

$$\text{Pr}(\text{Not}\;\text{Flowerin}{\text{g}}_{\mathrm{time}\;t}|\mathrm{Flowere}{\text{d}}_{\mathrm{time}\;t-1}\;\text{and}\;\text{Not}\;\text{dorman}{\text{t}}_{\mathrm{time}\;t}).$$

Our sample for retrogression was therefore set by the number of plants that flowered the previous year (time *t* − 1) that emerged as either flowering or non-flowering the next year (time *t*).

Growth and vital rate analyses are conducted in R 3.1.0 (R Development Core Team 2014) using the *lme4* package (Bates *et al.* 2014). To account for repeated measures and blocking effects, we include random intercepts for individual plants and pairs of treatments within a plot. For each response variable we test for significant differences between annual means using the *multcomp* package in R (Bretz *et al.* 2010). We test for the presence of a long-term trend since 2006 in each treatment mean by specifying a trend contrast (Rosenthal and Rosnow 1985; Gurevitch and Chester 1986). All tests are planned contrasts so we do not correct for multiple comparisons. To further investigate trends in flowering frequencies, we also analyse these data using a two-level hierarchical model with time as a continuously varying main effect and year as a random effect.

Results of flowering and retrogression analyses are reported as effect sizes using odds ratios (OR) (Rita and Komonen 2008). Odds ratios have a lower bound of zero and no upper bound. Odds ratios of 1 indicate no difference between two treatments in the odds of an event happening. Statistical tests for OR therefore test whether they are different from 1. Odds ratios and their 95 % confidence intervals (CIs) are given in the text on their normal scale but graphed on a log scale to improve interpretation (*sensu* Galbraith 1988).

#### Mark-recapture models

We use mark-recapture models, a modified logistic regression approach (Kéry *et al.* 2005), to estimate the probability of prolonged vegetative dormancy. To test for pre-existing differences in dormancy rates, we conduct separate mark-recapture analyses of the 3 years prior to implementation of the removal treatment (2003–05) and the 7 years after the treatment began (2007–13). Mark-recapture results are assessed using the small sample size corrected information criteria AICc (AICc = AIC + 2*k*(*k*+1)/(*n*−*k*−1), where *k* = the number of parameters and *n* = sample size) to rank the explanatory ability of different models (Anderson 2010). To summarize the data we also analyse the entire data set (2003–13) and calculate the mean difference in dormancy rates between treatments. We first calculate dormancy rates for each treatment in each year, calculate the difference between these means and average the differences for the pre- and post-treatment time periods. We use the delta method (Powell 2007) in the R package *msm* to combine multiple standard errors and construct 95 % CIs around our final effect size estimates. Mark-recapture models are run in the R package *marked* (Laake *et al.* 2013).

#### Missing data due to herbivory

Deer browse compromised our ability to gain information on some individuals. Deer preferentially browse flowering *Maianthemum* and flowering individuals are of larger size than non-flowering individuals (N. L. Brouwer and S. Kalisz, unpubl. data). Accordingly, in the cases where an individual was browsed before its reproductive status was determined during the 10 annual censuses (*n* = 103 instances across 10 years), we assumed the browsed individual was flowering. Further, if browse occurred before an individual's size data was collected or size was otherwise unavailable, we used linear imputation (Gelman and Hill 2006) to estimate its size (412 instances of size imputation out of 1481 total size records). Including imputed size data for the browsed plants prevents biasing our results against detecting a treatment effect (Hadfield 2008; Nakagawa and Freckleton 2008).

We imputed missing size data using estimates generated from multiple rounds of linear regression based on observed size data from the years prior to and after the missing data. We averaged these multiple estimates to arrive at a final imputed size estimate for each browsed individual. Linear regression models included all available covariates, including previous size, current status, treatment and reproductive output for flowering plants. We validated our imputations by comparing mean plant size and the overall size distribution in the population with and without imputed data **[see Supporting Information—Table S1]**.

## Results

### Greenhouse study: assessing potential for carbon stress

**Figure 1.**
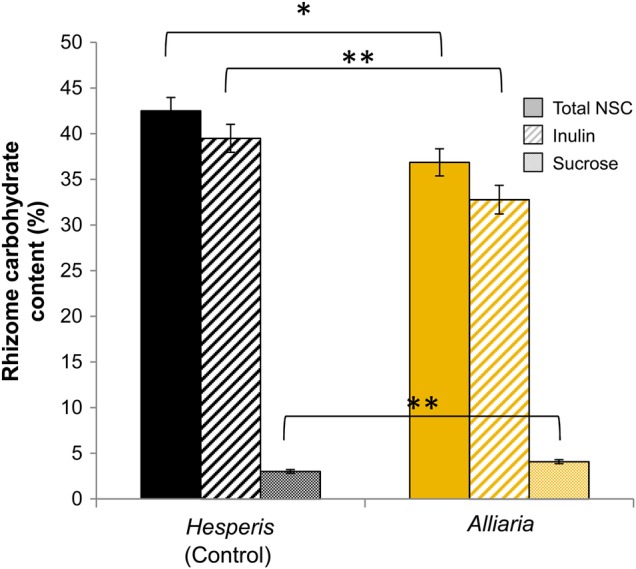
*Maianthemum racemosum* rhizome carbohydrate content (%) from *Alliaria* (yellow) and *Hesperis* (control; black) treatments in the greenhouse experiment. Total NSC content is shown in solid-coloured bars. Total NSC is a composite measure of stored sugars (inulin; bars with diagonal shading) and mobile sugars (sucrose; stippled bars). Values are least squares means from ANCOVAs ±1 standard error. *P < 0.05; **P < 0.005.

All *M. racemosum* plants examined exhibit colonization by internal RFS structures. However, *Maianthemum*'s rhizome carbohydrates were significantly affected by the *Alliaria* treatment (MANCOVA; Roy's greatest root = 7.57, *P* = 0.002), with plants in the *Alliaria* treatment experiencing a significant reduction in total NSC (Fig. 1; ANCOVA *F*_1,36_ = 7.31, *P* = 0.01). Specifically, plants treated with *Alliaria* stored, on average, 17 % less inulin relative to plants in the *Hesperis* treatment (Fig. 1; ANCOVA *F*_1,36_ = 9.28, *P* = 0.004). While plants in the *Alliaria* treatment had fewer stored sugars, they had higher sucrose concentrations in their rhizomes compared with plants in the *Hesperis* treatment (Fig. 1; ANCOVA *F*_1,36_ = 12.88, *P* = 0.001). The increase in mobile sugars did not compensate for the dramatic difference in stored sugars between treatments as total NSC in the *Alliaria*-treated plants was 13 % lower than that of *Hesperis*-treated plants. Harvest date was not a significant predictor of total NSC, inulin or sucrose.

### Field study: impact on vital rates

#### Growth

**Figure 2.**
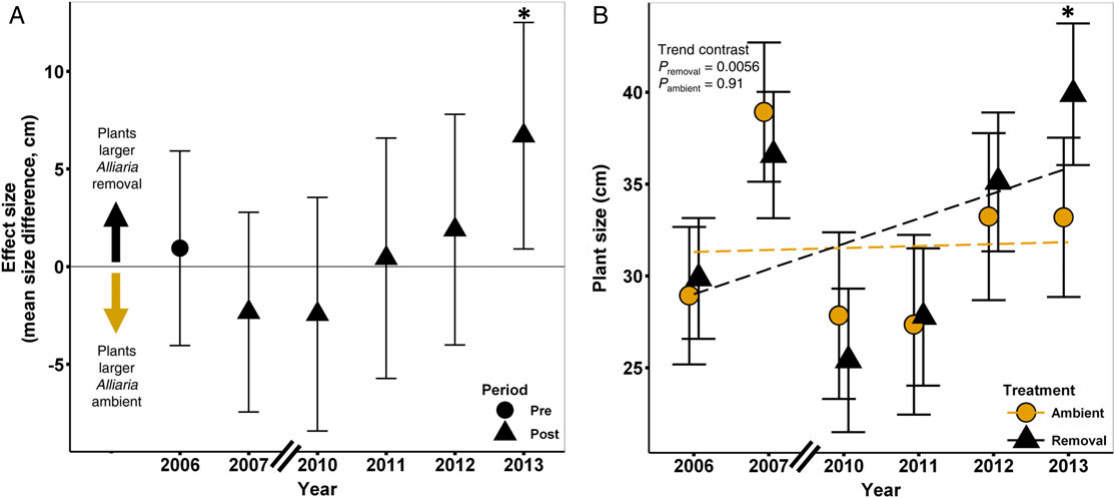
Effect of *Alliaria* on plant size of *Maianthemum* marked in the initial 2003 survey of the field experiment. (A) Mean difference (effect size) in plant size between *Alliaria* in ambient and removal treatments. (B) Annual mean plant sizes in both treatments and ANOVA trend contrasts. Error bars represent ±95 % CIs. Asterisk indicates a significant difference in plant size between the two treatments (*P* < 0.05). Size data were not available for 2008 and 2009.

Prior to implementation of the removal treatment, there was no difference in the mean size of plants in the initial 2003 cohort (Fig. 2; *P* = 0.55). By 2013 plants in the removal treatment are significantly larger than those in the ambient *Alliaria* treatment (mean difference = 6.70 cm, SE = 2.96; *P* = 0.02). There is a significant positive linear trend in size from 2006 to 2013 (trend contrast *P* = 0.0056) in the *Alliaria* removal plots but no trend in the ambient plots (*P* = 0.91).

#### Flowering

**Figure 3.**
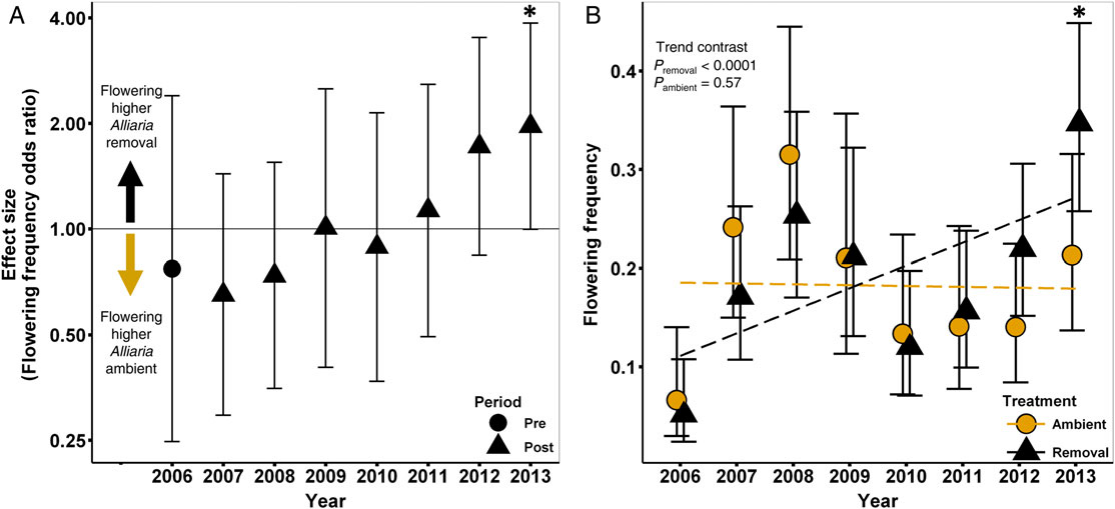
Effect of *Alliaria* on *Maianthemum* flowering frequency. (A) Mean difference (effect size, ES) in flowering frequency in *Alliaria-*ambient and removal plots. Effect size is expressed as an OR and plotted on the log scale. (B) Annual mean flowering frequencies for both treatments and ANOVA trend contrasts. Error bars represent ±95 % CIs. Asterisk indicates a significant effect of *Alliaria* removal (*P* < 0.05).

There is no significant difference in flowering probability across treatments for the first 6 years of the *Alliaria* removal (e.g. Fig. 3; *P*_2006_ = 0.65, *P*_2007_ = 0.29, *P*_2008_ = 0.42). However, by 2012 the flowering probability is ‘leaning’ (*sensu* Tukey 1991) in the predicted direction (OR = 1.72, CI_95%_ = 0.84–3.52, *P* = 0.14) and by 2013 is significantly higher (OR = 1.96 CI_95%_ = 1.0–3.87, *P* = 0.051) in the removal treatment. Across all years (2006–13) there is an increasing trend in flowering probability in the removal treatment (trend contrast *P* = 0.00008) but no increase in the ambient treatment (*P*_trend_ = 0.57).

Analyses using time as a continuous variable and year as a random effect confirmed that flowering frequencies diverged between the treatments (treatment × time *χ*^2^ = 6.81, *P* = 0.009) with a significant positive linear trend in the removal treatment (*β*_removal × time_ = 0.18, SE = 0.069) contrasted with evidence of a decrease in flowering probability in *Alliaria*-ambient plots (*β*_time_ = −0.10, SE = 0.072).

#### Retrogression

The number of flowering individuals was too low in 2005 and 2006 to accurately estimate retrogression of flowering plants in 2006 and 2007. By 2011, there was evidence that removal-treatment plants were less likely to retrogress (OR = 0.28 CI_95%_ = 0.052–1.57, *P* = 0.15) and in 2012 they were significantly less likely to retrogress (OR = 0.14 CI_95%_ = 0.021–0.96, *P* = 0.045). There was a significant decreasing trend in retrogression in the removal treatment from 2008 until 2013 (*P*_trend_ = 0.011) but no trend in the ambient treatment (*P*_trend_ = 0.90).

#### Dormancy

Dormancy rates were highly variable between years, ranging from <10 to >30 %, but estimated to be lower in the *Alliaria* removal treatment in six out of 7 years **[see Supporting Information—Table S2]**. For years prior to the implementation of the *Alliaria* removal treatment (2003–06) the best-ranked model contains only a year effect (Table 1) while for models of post-treatment years (2007–13) and the entire dataset (2003–13) the best models contain an effect of *Alliaria* removal, indicating that dormancy rates were typically lower in this treatment. There was an initially large difference in dormancy rates between plots that would be allocated to the two treatments in the first year of the study **[see Supporting Information—Table S2]**, potentially resulting in the model of the pre-treatment years containing an *Alliaria* removal effect (AICc = 454.6) ranked almost as high as a year-only model (AICc = 452.8). However, since the year-only model has a lower AICc and fewer parameters, the larger model is not considered competitive (Arnold 2010). Moreover, in the other two pre-treatment years (2004 and 2005), there is no difference between dormancy estimates **[see Supporting Information—Table S2]**. The results of model selection are reinforced by the calculation of average effect sizes for the period prior to *Alliaria* removal and after removal (Fig. 5). Prior to removal there is no significant difference between dormancy rates (ES = −0.05, CI_95%_ = −0.13–0.03) but after removal dormancy rates are ∼7 % lower than in the *Alliaria*-ambient treatments (ES = −0.069, CI_95%_ = −0.12 to −0.2).

**Table 1.** Ranking of mark-recapture models testing the effects of *Alliaria* removal on prolonged vegetative dormancy. Three sets of models were run over different time periods during the study: Set 1: years before *Alliaria* removal began (Pre-treatment); Set 2: years after the annual weeding treatment was initiated (post-treatment) and Set 3: all years. *N*, number of plants tracked over each time period; *K*, number of parameters in a model; Ln(lik), log likelihood. To calculate the mean pre-treatment and post-treatment effect size (Fig. 5) we used the parameters from the ‘Removal × Year’ model in the ‘All years’ model Set 3.

Set	Period	Model	*N*	*K*	AICc	ΔAICc	Ln(lik)
1	Pre-*Alliaria* removal (2003–06)	Year	158	5	452.8	0.00	−216.21
		Removal + Year		6	454.6	1.74	−215.00
		Removal × Year		9	466.2	11.59	−214.47
2	Post-*Alliaria* removal (2007–13)	Removal + Year	210	9	1166.4	0.00	−564.73
		Year		8	1172.4	6.03	−569.84
		Removal × Year		15	1187.2	14.76	−562.34
3	All years (2003–13)	Removal + Year	236	12	1646.3	0.00	−798.46
		Year		11	1652.5	6.23	−803.68
		Removal × Year		21	1680.3	27.74	−795.98

## Discussion

To our knowledge this is the first study to explore the connections between an allelopathic invasive species' impacts on the soil biotic environment and changes in individual plants' carbon status and vital rates. The results presented here in conjunction with prior studies substantiate multiple steps in a physiologically based causal pathway between invasion and population-level impacts on native plants. Our prior work demonstrates that *Alliaria* treatment of soil around *Maianthemum* reduces the density of soil fungal hyphae (A. N. Hale *et al.*, submitted for publication) and plant photosynthetic rates (Hale *et al.* 2011). Here, our results demonstrate that treatment with *Alliaria* across the entire growing season results in negative effects on season-long carbon storage (Fig. 1). Relative to control plants, *Maianthemum* exposed to *Alliaria* stored 17 % less inulin in their rhizomes and experienced an overall reduction in total NSCs at the end of the season. Stomatal conductance modulates carbon fixation and is a key physiological rate affected by *Alliaria* exposure (Hale *et al.* 2011). Interestingly, a recent meta-analysis (Augé *et al.* 2014) comparing the effects of AMF inoculation on stomatal conductance (*g_s_*) in field vs. greenhouse studies indicates that greenhouse experiments have smaller effect sizes than field studies. Thus, our carbon storage results are likely conservative estimates of the carbon impacts of mutualism disruption in the field.

**Figure 4.**
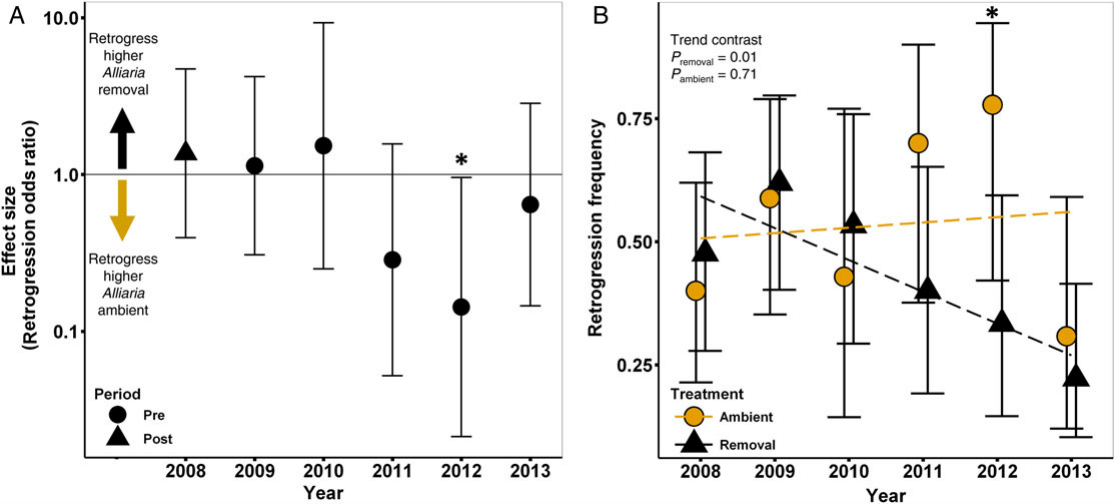
Effect of *Alliaria* on *Maianthemum* retrogression from flowering to non-flowering. (A) Annual mean difference in retrogression frequency (ES) in *Alliaria*-ambient and removal plot. Effect size is expressed as an OR and plotted on the log scale. (B) Mean retrogression frequencies in both treatments and ANOVA trend contrasts. Error bars represent ±95 % CIs. Asterisk indicates a significant effect of *Alliaria* removal (*P* < 0.05). Retrogression is calculated conditional on a plant being observed above-ground and not dormant. Sample sizes for 2006 and 2007 were insufficient for vital rate calculation.

**Figure 5.**
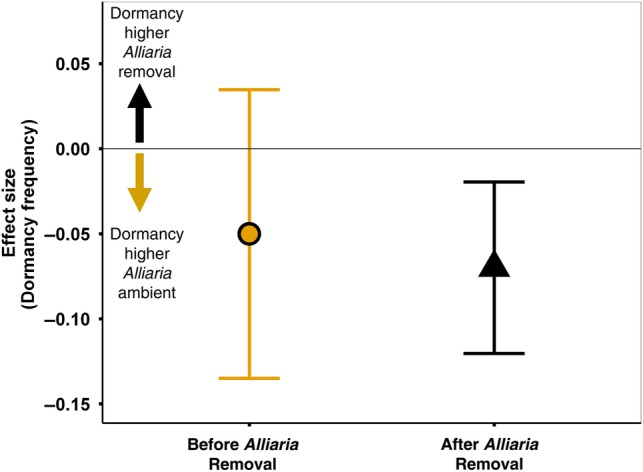
Effect size of *Alliaria* removal on the frequency of prolonged vegetative dormancy in *Maianthemum* before (2003–06; yellow) and after the treatment began (2007–13; black). Calculated with mark-recapture models; error bars represent ± 95 % CIs. Asterisk indicates a significant effect of *Alliaria* removal (*P* < 0.05).

Over time, chronic exposure to *Alliaria* was predicted to compound this carbon deficit and affect plant growth and vital rates. Results from our long-term field study of *Alliaria* removal are consistent with this prediction. Individual aboveground plant size (Fig. 2) and multiple carbon-intensive and size-dependent vital rates (Figs 3–5) are positively affected in *Alliaria* removal relative to *Alliaria*-ambient plots.

Other experimental studies where *Alliaria* and native plants are grown together in pots (Meekins and McCarthy 1999; Wixted and McGraw 2010; Lankau 2012; Smith and Reynolds 2014) or in the field (McCarthy 1997; Carlson and Gorchov 2004; Cipollini *et al*. 2008; Lankau 2011*b*) also find negative effects of *Alliaria* on native species. Competition, direct allelopathic phytotoxicity and allelopathic RFS-mutualism disruption are all mechanisms that could contribute to these results. Our greenhouse experiment adds support to the idea that it is *Alliaria*'s disruption of key belowground mutualists (RFS) rather than competition or direct phytotoxicity that accounts for its success as an invader. Below we discuss the general support or lack thereof for the likelihood of all three mechanisms.

### Competition

We are aware of only two studies that have attempted to quantify reciprocal competition between *Alliaria* and focal plants. These pot studies found that *Alliaria* was equal to or weaker in competitive ability than three of four species tested (Meekins and McCarthy 1999; Leicht-Young *et al*. 2012). However, these studies are problematic in that they cannot separate competition from phytotoxicity or mutualism disruption. Bossdorf *et al*. (2004) found that *Alliaria* individuals from the native range outcompete *Alliaria* plants from the invaded range, supporting the hypothesis that invasive *Alliaria* express a different trade-off relative to their source populations. Invasive *Alliaria* are armed with novel allelochemical weapons but have evolved to be less competitive (Bossdorf *et al*. 2004). Further, field experiments demonstrate that native competitors can suppress *Alliaria* performance and abundance when the natives are not experiencing overabundant herbivore pressure (Eschtruth and Battles 2009*a*), as deer preferentially consume native plants and facilitate the high population growth and spread of *Alliaria* (Kalisz *et al.* 2014). In experimental studies that exclude deer from invaded sites, *Alliaria* abundance rapidly declines (Eschtruth and Battles 2009*b*; Knight *et al*. 2009; Kalisz *et al*. 2014). In total, these results underscore the widely held view that *Alliaria* is a relatively poor competitor (Rodgers *et al.* 2008*a*).

### Direct phytotoxicity

Glucosinolates are known antimicrobial chemicals produced by members of the mustard family as defences against pathogens (Tierens *et al.* 2001). While *Alliaria*'s allelochemicals can be inhibitory to germinating seeds and inhibit new seedling root growth (lettuce and radish seed experiments: Vaughn and Berhow 1999; Roberts and Anderson 2001; Pisula and Meiners 2010; *Impatiens* and *Viola* seed experiments: Prati and Bossdorf 2004; Barto *et al*. 2010; Cipollini and Flint 2013), to our knowledge direct toxicity of *Alliaria* on mature plant tissues has never been demonstrated. *Alliaria* invades forest understories dominated by adult perennial plants dependent on RFS. The direct effect of allelochemicals is inversely proportional to target plant density or biomass (Weidenhamer 2006). Single-celled fungal spores and thin fungal hyphae should be much more susceptible to *Alliaria* allelochemicals than mature plant tissues. Thus, while we cannot rule out direct phytotoxic effects of *Alliaria* on adult *Maianthemum* performance in our field or greenhouse experiments, a direct allelochemical effect is likely of small magnitude relative to indirect effects on RFS.

### RFS-mutualism disruption

Mounting evidence shows that *Alliaria* can exert potent indirect effects on plants by suppressing RFS. Glucosinolates, like those produced by *Alliaria*, have a short half-life in the soil (<15 h; Gimsing *et al.* 2006). Yet, native plants grown in soils conditioned by *Alliaria*, treated with *Alliaria* tissue extracts, or collected from *Alliaria*-invaded sites all express reduced growth (Stinson *et al.* 2006; Callaway *et al.* 2008; Wolfe *et al.* 2008) despite the fact that the volatile allelochemicals were likely no longer present. Importantly, these studies demonstrate that *Alliaria* impacts are similar in magnitude to soil sterilization and that experimental soils result in lower colonization of roots by mycorrhizae (Stinson *et al.* 2006; Callaway *et al.* 2008; Wolfe *et al.* 2008). Finally, *Maianthemum* plants treated with *Alliaria* retain RFS structures internal to their roots, while exhibiting significant declines in soil hyphae (A. N. Hale *et al.*, submitted for publication). Together these experiments provide strong support for RFS-mutualism disruption and that its effects are of large magnitude relative to competition or direct phytotoxicity.

Mechanistically, our working model linking RFS-mutualism disruption to carbon stress is based on the following premises: If *Alliaria*'s allelochemicals destroy the hyphal network, yet the normally long-lived internal structures (Brundrett and Kendrick 1990) remain intact, then we would predict that the plant would increase carbon allocation to its RFS to provision the regrowth of the soil hyphal network, resulting in significant carbon stress for the plant. Loss of the hyphal network severely limits available soil nutrients and water to the plant (Newsham 2011; Augé *et al*. 2014). As a result, the plants photosynthesize less (Hale *et al*. 2011) and fix less carbon (NSC; Fig. 1). With this limited carbon pool, we suggest that plants may maintain concentrations of mobile sugars in the rhizome and roots to re-establish a functional RFS hyphal network that is repeatedly destroyed by our application of fresh *Alliaria* tissue. While our results are consistent with this working model (e.g. we observe greater sucrose concentrations in the rhizome of *Alliaria* vs. *Hesperis*-treated plants (Fig. 1)), additional experiments are needed to fully explore this hypothesis.

We note that the effects of allelopathic mutualism disruption by *Alliaria* could be amplified by additional factors. Like other invasive species of deciduous forests (Ehrenfeld *et al.* 2001; Poulette and Arthur 2012; Smith and Reynolds 2012; Kuebbing *et al.* 2014; Schuster and Dukes 2014), *Alliaria* can affect multiple components of the soil environment. *Alliaria* increases soil nutrient availability (Rodgers *et al.* 2008*b*), litter decomposition rates and nitrogen loss (Ashton *et al.* 2005). Since the RFS community in general (Van Diepen *et al*. 2011) and specific RFS–plant interactions (e.g. Klironomos 2002) are sensitive to soil conditions, multiple invader-mediated changes to the soil environment could magnify the impacts of allelopathic RFS-mutualism disruption. These diverse and widespread consequences of invasive species for soil environments and RFS communities are alarming given the potentially central role RFS and other microbes play in the diversity, productivity and functioning of plant communities (van der Heijden *et al*. 2008).

Our greenhouse study indicates that *Maianthemum* carbon storage declines significantly in response to *Alliaria* treatment in just one growing season. In contrast, we observe a relatively slow recovery of individual size, growth and vital rates following *Alliaria* removal in our field study. The predicted significant trends indicative of recovery (Figs 2–4) emerged after a few years of *Alliaria* removal while significant differences within the single-year comparisons were not seen until ∼6–7 years post removal (2012 or 2013). Two, non-mutually exclusive mechanisms could underlie this lag. First, the lag could be due to *Maianthemum*'s habit (LaFrankie 1985). In general, forest understory herbaceous perennials are light-limited, slow-growing, long-lived species (Whigham 2004) with slow responses to perturbation (Morris *et al.* 2008). Our data are consistent with the idea that following *Alliaria* removal, *Maianthemum* may take multiple years to re-gain sufficient carbon stores to allow size growth, sustain flowering and maintain low dormancy rates. Second, the observed lag in *Maianthemum* vital rate responses may be due to slow recovery of the RFS soil community following *Alliaria* removal, a phenomenon observed by Anderson *et al.* (2010) and Lankau *et al.* (2014). If populations of beneficial RFS have gone locally extinct and low dispersal distance limits RFS re-colonization (Rout and Callaway 2012), then the observed time lag of *Maianthemum* could be due to the low abundance of effective fungal partners. Given the reciprocal obligate dependence of AMF and forest herbaceous perennial plants, declines in the native understory community may drive reciprocal declines in the RFS soil community (Lankau *et al.* 2014).

## Conclusions

Increases in invasive species are generally correlated with declines in native biodiversity (e.g. Butchart *et al.* 2010). However, the mechanistic underpinnings leading to native population collapse are rarely understood yet are the subject of numerous studies and invasion hypotheses (Levine *et al*. 2003; Hulme *et al*. 2013). The disruption of plant soil feedbacks and root fungal symbioses are common aspects of plant invasions (i.e. Grove *et al*. 2012; Meinhardt and Gehring 2012; Ruckli *et al.* 2014; Shannon *et al.* 2014). As suggested by Hale and Kalisz (2012), chronic RFS-mutualism disruption could act as the first step in native plant biodiversity loss. In our system, the disruption of RFS by an allelopathic invader appears to begin a downward spiral in the physiological function (Hale *et al*. 2011), carbon status (Fig. 1) and ultimately vital rates (Figs 2–5) of a common native forest plant. Loss of these critical belowground mutualisms may be the proximate cause of plant mortality that is instead attributed to second-order effects (e.g. drought or herbivory) that are easier to observe (*sensu* McDowell 2011). Additional studies in invaded communities that explore the links between plant physiology, carbon allocation and population demographic performance are needed to determine the generality of these results. Mutualism disruption may be a widespread mechanism that helps explain how invasive species can cause large-scale changes to forest biodiversity observed in the wake of invasion (e.g. Rodgers *et al.* 2008*a*).

## Sources of Funding

Funding was supplied by a United States National Science Foundation award DEB-0958676 to S.K., a NSF pre-doctoral fellowship to N.L.B. and a Phipps Conservatory Botany-in-Action award and an Andrew K. Mellon pre-doctoral fellowship to A.N.H.

## Contributions by the Authors

S.K. conceived, designed, implemented and led data collection of the field experiment and assisted with the conception, design and implementation of the greenhouse experiment. A.N.H. designed, implemented and analysed data from the greenhouse experiment and assisted in data collected for the field experiment from 2008 to 2011. N.L.B. managed and analysed data from the field experiment and assisted with data collection for the field experiment since 2010. All three authors collaborated on the conception and writing of this article.

## Conflicts of Interest Statement

None declared.

## Supporting Information

The following additional information is available in the online version of this article –

**Table S1**. Validation of imputed *Maianthemum* size data from field experiment. Original and imputed size data are compared using *t*-tests and Kolmogorov–Smirnov tests.

**Table S2.** Estimated frequency of prolonged vegetative dormancy of *Maianthemum* from field experiment using a Mark-Recapture model.

Additional Information
