# Supplementary material for: Mutualism-disrupting allelopathic invader drives carbon stress and vital rate decline in a forest perennial herb
Source: AoB Plants. 2015 Feb 27;7:plv014. doi: 10.1093/aobpla/plv014 (PMC4374104; doi:10.1093/aobpla/plv014)
Supplement: Additional Information [file supp_7_plv014_index.html]

Mutualism-disrupting allelopathic invader drives carbon stress and vital rate decline in a forest perennial herb — Additional Information 

# Mutualism-disrupting allelopathic invader drives carbon stress and vital rate decline in a forest perennial herb

## Additional Information

Additional Information

**Files in this Data Supplement:**

- Supplementary Table 1 - Doc file
- Supplementary Table 1 - Doc file
