# Supplementary material for: Mutualism-disrupting allelopathic invader drives carbon stress and vital rate decline in a forest perennial herb
Source: AoB Plants. 2015 Feb 27;7:plv014. doi: 10.1093/aobpla/plv014 (PMC4374104; doi:10.1093/aobpla/plv014)
Supplement: Additional Information [file supp_plv014_plv014supp_table1.docx]

Table S1: Validation of imputed *Maianthemum racemosum* size data. Imputation was done for plants with missing size data due to deer browse (primarily on flowering individuals) and for plants from the two years (2008-2009) when sizes could not be collected (n=412 intances). T-tests were conducted on log-transformed data. Kolmogorov-Smirnov (KS) test indicates whether two samples come from the same distribution. Imputation of size data increases the sample size of flowering plants by 319, which increases the overall mean plants size by 2.2 cm. Within life history stages (non-flowering and flowering) there are no significant differences between the original and imputed data.

| **Plant** | **Original Data** | | **Imputed Data** | | **t-test** | | **KS-test** | |
| --- | --- | --- | --- | --- | --- | --- | --- | --- |
| **Status** | **Mean (SE)** | **N** | **Mean (SE)** | **N** | **t** | **p** | **D** | **p** |
| All plants | 28.8 (0.5) | 963 | 31.0 (0.4) | 1481 | 3.9 | <0.001 | 0.07 | 0.004 |
| Non-flowering | 24.5 (0.3) | 808 | 24.8 (0.3) | 1127 | 1.4 | 0.2 | 0.06 | 0.1 |
| Flowering | 51.5 (1.2) | 155 | 51.0 (0.7) | 354 | 0.01 | 1 | 0.07 | 0.7 |
