# Supplementary material for: Mutualism-disrupting allelopathic invader drives carbon stress and vital rate decline in a forest perennial herb
Source: AoB Plants. 2015 Feb 27;7:plv014. doi: 10.1093/aobpla/plv014 (PMC4374104; doi:10.1093/aobpla/plv014)
Supplement: Additional Information [file supp_plv014_plv014supp_table2.docx]

Table S2: Estimated frequency of prolonged vegetative dormancy in plots allocated to *Alliaria* Ambient and Removal treatments, in years prior to (2004-2006) and after (2007-2013) implementation of the treatment. Estimate are from a mark-recapture model using all years of data and a Removal*Year interaction (Model set 3, Table 1). Period indicates years before or after implementation of the *Alliaria* removal treatment.

|  |  | ***Alliaria* Ambient Plots** | | | ***Alliaria* Removal Plots** | |
| --- | --- | --- | --- | --- | --- | --- |
| **Period** | **Year** | **Frequency** | **SE** |  | **Frequency** | **SE** |
| Pre-*Alliaria* | 2004 | 0.31 | 0.078 |  | 0.18 | 0.061 |
| Removal | 2005 | 0.11 | 0.052 |  | 0.10 | 0.040 |
|  | 2006 | 0.08 | 0.039 |  | 0.07 | 0.032 |
|  |  |  |  |  |  |  |
| Post-*Alliaria* | 2007 | 0.14 | 0.046 |  | 0.09 | 0.035 |
| Removal | 2008 | 0.18 | 0.051 |  | 0.09 | 0.034 |
|  | 2009 | 0.51 | 0.065 |  | 0.38 | 0.055 |
|  | 2010 | 0.25 | 0.054 |  | 0.13 | 0.035 |
|  | 2011 | 0.25 | 0.056 |  | 0.12 | 0.035 |
|  | 2012 | 0.15 | 0.047 |  | 0.13 | 0.036 |
|  | 2013 | 0.09 | 0.049 |  | 0.13 | 0.043 |
